# Comparison between meropenem and ceftolozane/tazobactam: possible influence of CRRT

**DOI:** 10.1186/s13054-021-03837-6

**Published:** 2022-01-07

**Authors:** Patrick M. Honore, Sebastien Redant, Thierry Preseau, Sofie Moorthamers, Keitiane Kaefer, Leonel Barreto Gutierrez, Rachid Attou, Andrea Gallerani, Willem Boer, David De Bels

**Affiliations:** 1 ICU Department, Centre Hospitalier Universitaire Brugmann-Brugmann University Hospital, Place Van Gehuchtenplein, 4, 1020 Brussels, Belgium; 2 ED Department, Centre Hospitalier Universitaire Brugmann, Brussels, Belgium; 3 ICU Department, Centre Hospitalier Universitaire Brugmann, Brussels, Belgium; 4 Intensive Care Department, Ziekenhuis Oost Limburgh, Campus St Jan, Genk, Belgium

**Keywords:** Ventilator hospital-acquired bacterial pneumonia, Ceftolozane/tazobactam, meropenem, CRRT, Confounders

In their recent study [1], Timsit et al. conclude that mortality risk with ventilator hospital-acquired bacterial pneumonia (vHABP) was over twice as high when treated with meropenem compared to ceftolozane/tazobactam (C/T). However, the percentage of patients in the database with vHABP who had a creatinine clearance (CrCl) between 15 and 30 ml/min was 12% in both groups [1]. Of these, around 40% had a sequential organ failure assessment (SOFA) score > 7 with vasopressor use in more than 50% in both groups. Consequently, it is reasonable to assume that most of these patients were undergoing renal replacement therapy (RRT), most likely continuous RRT (CRRT) though this was not reported [1]. While a dose of C/T of 3 gr (2 g ceftolozane and 1 g tazobactam) three times a day will surely be above the minimal inhibitory concentration (MIC) most of the time even on CRRT [2], this is not the case for meropenem 1 gr three times a day, as in a number of cases this dose will fall below the MIC when undergoing CRRT [1]. Kothekar et al. concluded that in septic shock patients, extended infusions (EI) of 1000 mg of meropenem over 3 h, administered every 8 h, provided adequate coverage against sensitive strains of Enterobacteriaceae, *Pseudomonas aeruginosa* and *Acinetobacter baumannii* [3]. However, this dosing regimen failed to achieve a fraction of time (fT) > 4 μg/mL > 40 for activity against more resistant strains of these organisms in more than one-third of patients [3]. A bolus of 500 mg followed by EI of 1500 mg every 8 h was predicted to achieve this target in all patients [3]. If drug dose adaptation was not adhered to in CRRT patients and continuous infusion (CI) not used in cases of pathogens with a MIC ≥ 4, as recommended [4] some patients may have been underdosed, even with 1 g every 8 h [3, 4], as meropenem is significantly eliminated by CRRT [4]. In addition, in the same study adjunctive therapy with amikacin 15 mg/kg was permitted for the first 72 h of study treatment where ≥ 15% of *Pseudomonas aeruginosa* were known to be meropenem resistant [1]. Under CRRT, the recommended dose of amikacin to avoid failure is 25 mg/kg [5]. In conclusion, underdosing of antibiotics in patients undergoing CRRT may go some way to explaining the findings reported by Timsit et al.

## Data Availability

Not applicable.

## Abbreviations

vHABP: Ventilator hospital-acquired bacterial pneumonia
C/T: Ceftolozane/tazobactam
ClCr: Creatinine clearance
SOFA: Sequential organ failure assessment
RRT: Renal replacement therapy
Continuous RRT: Continuous renal replacement therapy
MIC: Minimal inhibitory concentration
EI: Extended infusions
fT: Fraction of time
CI: Continuous infusion
