# Supplementary material for: Comparison between meropenem and ceftolozane/tazobactam: possible influence of CRRT
Source: Crit Care. 2022 Jan 7;26:15. doi: 10.1186/s13054-021-03837-6 (PMC8742339; doi:10.1186/s13054-021-03837-6)
Supplement: Related texts

# Reply: Authors Response

**Authors:** Jean‑François Timsit, Jennifer A. Huntington, Richard G. Wunderink, Marin H. Kollef, Ignacio Martin‑Loeches, Brian Yu, Erin H. Jensen, Dominik J. Wolf, Christopher J. Bruno

**Affiliations:** 5 grid.508487.6 0000 0004 7885 7602 Intensive Care Medicine Department, Université Paris Diderot, Paris, France; 6 grid.417993.1 0000 0001 2260 0793 MRL, Merck & Co., Inc., 2000 Galloping Hill Road, Kenilworth, NJ 07033 USA; 7 grid.16753.36 0000 0001 2299 3507 Pulmonary and Critical Care Division, Northwestern University Feinberg School of Medicine, Chicago, IL USA; 8 grid.4367.6 0000 0001 2355 7002 Division of Pulmonary and Critical Care Medicine, Washington University School of Medicine, St. Louis, MO USA; 9 grid.416409.e 0000 0004 0617 8280 Department of Intensive Care Medicine, Multidisciplinary Intensive Care Research Organization (MICRO), St James’ Hospital, Dublin, Ireland; 10 Hospital Clinic, Universitat de Barcelona, IDIBAPS, CIBERES, Barcelona, Spain

**Dear Editor,**

We appreciate the letter from Honore et al. expressing concern about the potential for underdosing meropenem in participants who may have received renal replacement therapy (RRT) in our study [1]. Honore et al. assumed that because 40% of participants in our subgroup analysis had sequential organ failure assessment scores > 7 and ≈50% received concomitant vasopressors, most would have undergone continuous RRT (CRRT). However, this was not the case.

Per the study protocol, any requirement for peritoneal dialysis or hemodialysis or hemofiltration were exclusion criteria and RRT was not permitted during study treatment. Any participant who developed creatinine clearance (CrCl) < 15 mL/min or was placed on RRT was required to be withdrawn from randomized study treatment and switched to standard-of-care antibacterial therapy, because optimal dosing recommendations for participants with renal impairment receiving RRT had not been determined for ceftolozane/tazobactam or meropenem at the time the study was conducted. The current meropenem label still lacks dosing recommendations for patients undergoing RRT [6]. We agree with Honore et al. that inclusion of participants receiving RRT in the meropenem arm would have been of concern.

Recently, several studies have been conducted to determine optimal dosing of ceftolozane/tazobactam in adults with hospital-acquired bacterial pneumonia/ventilator-associated bacterial pneumonia (HABP/VABP) and renal impairment or augmented renal clearance (ARC). The results suggest that the following ceftolozane/tazobactam doses, administered every 8 h according to renal function, are recommended for adults with HABP/VABP [7–9]: CrCl > 50 mL/min (including critically ill patients with ARC): 3 g; CrCl 30 to ≤ 50 mL/min: 1.5 g; CrCl 15 to < 30 mL/min: 750 mg; and end-stage renal disease on hemodialysis: single loading dose of 2.25 g, followed by 450 mg every 8 h.

Importantly, among the ventilated HABP (vHABP) subgroup, only 1 of the 108 participants in the meropenem arm underwent RRT, including CRRT, while on study treatment (i.e., a protocol deviation). Thus, the influence of RRT on the analysis and interpretation of results reported for participants with vHABP treated with meropenem was exceedingly small. None of the 99 participants with vHABP in the ceftolozane/tazobactam treatment arm received RRT while on treatment.

In conclusion, RRT was not permitted during study treatment, and only 1 participant in the vHABP subgroup deviated from the protocol and received CRRT during study treatment. Therefore, underdosing of meropenem in the setting of RRT did not appreciably affect the findings of our recently published analysis of participants with vHABP [1].
